# Global treatment costs of breast cancer by stage: A systematic review

**DOI:** 10.1371/journal.pone.0207993

**Published:** 2018-11-26

**Authors:** Li Sun, Rosa Legood, Isabel dos-Santos-Silva, Shivani Mathur Gaiha, Zia Sadique

**Affiliations:** 1 Department of Health Services Research and Policy, London School of Hygiene and Tropical Medicine, London, United Kingdom; 2 Department of Non-communicable Disease Epidemiology, London School of Hygiene and Tropical Medicine, London, United Kingdom; 3 Department of Social and Environmental Health Research, London School of Hygiene and Tropical Medicine, London, United Kingdom; University of Florida, UNITED STATES

## Abstract

**Background:**

Published evidence on treatment costs of breast cancer varies widely in methodology and a global systematic review is lacking.

**Objectives:**

This study aimed to conduct a systematic review to compare treatment costs of breast cancer by stage at diagnosis across countries at different levels of socio-economic development, and to identify key methodological differences in costing approaches.

**Data sources:**

MEDLINE, EMBASE, and NHS Economic Evaluation Database (NHS EED) before April 2018.

**Eligibility criteria:**

Studies were eligible if they reported treatment costs of breast cancer by stage at diagnosis using patient level data, in any language.

**Study appraisal and synthesis methods:**

Study characteristics and treatment costs by stage were summarised. Study quality was assessed using the Drummond Checklist, and detailed methodological differences were further compared.

**Results:**

Twenty studies were included, 15 from high-income countries and five from low- and middle-income countries. Eleven studies used the FIGO staging system, and the mean treatment costs of breast cancer at Stage II, III and IV were 32%, 95%, and 109% higher than Stage I. Five studies categorised stage as in situ, local, regional and distant. The mean treatment costs of regional and distant breast cancer were 41% and 165% higher than local breast cancer. Overall, the quality of studies ranged from 50% (lowest quality) to 84% (highest). Most studies used regression frameworks but the choice of regression model was rarely justified. Few studies described key methodological issues including skewness, zero values, censored data, missing data, and the inclusion of control groups to estimate disease-attributable costs.

**Conclusions:**

Treatment costs of breast cancer generally increased with the advancement of the disease stage at diagnosis. Methodological issues should be better handled and properly described in future costing studies.

## Introduction

Breast cancer is the most common cancer among women worldwide, contributing more than 25% of the global new female cancer cases [1]. It is also the first leading cause of female cancer mortality, accounting for 14.7% of cancer deaths [1].

Breast cancer is a potentially curable disease if diagnosed and treated at an early stage. Surveillance, Epidemiology, and End Results (SEER) Program has reported that breast cancer cases diagnosed at an early stage (Stage I/II) have a better prognosis (5-year survival rate of 85%-98%). In contrast, patients diagnosed with advanced breast cancer (Stage III/IV) have a poor 5-year survival rate of 30%-70% [2]. Therefore, some intervention programmes have been initiated aiming for early diagnosis and treatment of breast cancer to reduce mortality and improve disease outcomes [3, 4].

Although the case for earlier diagnosis with respect to outcomes has been well made, the financial implications are less well understood [5, 6]. Stage of disease at diagnosis is an important predictor of treatment costs. Treatment for more advanced disease is often more intensive or invasive than treatment for the earlier stages [5]. As a result, a more advanced stage tends to be associated with more resource utilisation in addition to poorer health outcomes [7].

Treatment costs by stage at diagnosis are important in quantifying the gains from early detection. If early treatment lowers costs, this will help offset the cost of interventions for earlier diagnosis and treatment. In addition, treatment costs by stage would be valuable to inform the cost-effectiveness studies for treatment or preventative interventions of breast cancer. However, the mean costs by stage do not reveal the heterogeneity across patients. Patient level data can contain information such as socioeconomic group, medical history, and treatment options, thus allowing the comparison of costs across patient subgroups and identification of cost predictors. Therefore, availability of detailed patient level costing data by stage at diagnosis is important.

To date, no review has directly compared the methods used for collecting and analysing treatment costs of breast cancer across different settings. A systematic review, published in 2009, aimed to synthesize treatment costs of breast cancer per patient in the United States (US) [8]. However, this review did not assess between-study methodological differences, such as cost data collection methods, regression models, or whether breast cancer-attributable costs were estimated. Differences in methods should be examined, however, because they might have affected the cost estimates of breast cancer treatment.

In this paper, we undertook a systematic review of breast cancer treatment costs by stage at diagnosis based on patient level data to: (i) compare stage-specific treatment costs across countries at different levels of socio-economic development; and (ii) identify key methodological differences in costing approaches.

## Materials and methods

### Eligibility criteria

This study has been registered in PROSPERO international prospective register of systematic review (CRD42018097473). The inclusion criteria were based on the PICOS framework: (i) population: female breast cancer patients; (ii) intervention: any form of clinical treatment interventions; (iii) comparator: not restricted; (iv) outcome: direct medical treatment costs (inpatient and outpatient) by stage incurred in hospital settings at the patient level; and (v) study design: costing studies with primary data.

We excluded studies with the following characteristics: (i) no treatment cost estimates by stage; (ii) treatment costs not incurred in hospital settings which cannot reflect direct medical costs (inpatient and outpatient); (iii) costs not estimated from actual patient level data, but calculated according to treatment pathways in clinical guidelines; (iv) disease stages categorised neither as 0, I, II, III and IV in the International Federation of Gynaecology and Obstetrics (FIGO) staging system, nor as in situ, local, regional and distant cancer; and (v) review articles. Only studies that had primary data on the breast cancer costs were selected to avoid repeating previously published information. There was no language limit for the eligibility criteria.

### Search methods

We searched MEDLINE(R) (1946 to April Week 4 2018), EMBASE Classic + EMBASE (1947 to 30 April 2018), and NHS Economic Evaluation Database (NHS EED, 1960 to April 2018) with search terms in S1 Table. Also, reference lists from relevant primary studies and review articles were used to identify other relevant publications. Titles and abstracts were first reviewed, and full-texts of the studies that potentially met the eligibility criteria were retrieved and full-text reviewed.

### Data extraction

Two investigators independently extracted the study characteristics and treatment costs of breast cancer by stage at diagnosis. Most studies conducted cost analyses up to a specified time rather than over a lifetime horizon. Although some studies reported the annual costs, we extracted the cumulative costs during the pre-specified time horizons for comparative purposes. We first summarised the cumulative treatment costs of breast cancer patients by stage in all reviewed studies. Then we compared the costs in studies with the same pre-specified time horizons.

We used US dollars with the base year of 2015 to facilitate the comparison of costs. In this study, we used purchasing power parity (PPP) conversion factor to convert cost estimates reported in different currencies to US dollars, and used the consumer price index (CPI) for health care to convert cost estimates reported at different time points to the same year. PPP is the rate of currency conversion at which a given amount of currency will purchase the same volume of goods and services in two countries. CPI is a measure that examines the changes in the price level of a basket of consumer goods and services.

### Critical appraisal and methodological assessment

Two investigators used an established checklist by Drummond et al. [9] to assess the quality of reviewed studies independently. Items not applicable to costing studies were removed. A three-point response scale was added to better grade the quality of each item on the checklist, ranging from 0 (not considered), through 1 (partially considered), to 2 (fully considered) [10]. We summed up all scores and compared this with the maximum attainable score to calculate the percentage of the maximum attainable score.

In addition, we conducted a more detailed analysis of the methods used, including whether costs were based on charges or claims, the data collection approaches, use of control groups, descriptive analysis of mean costs by stage, regression model choices, censored data analysis, missing data analysis, and timing issues.

We distinguished between whether charges or claims were used because charges are often higher than the insurer claim costs [8], though either of which does not necessarily reflect the true economic costs of providing the medical services.

Costing data collection methods should depend on the aim of the study and the availability of data [11]. One method is the ingredient approach, also called micro-costing, with resources and the associated unit costs directly measured. At the other end of the spectrum is the gross costing or top-down method. In this approach, the costs are usually estimated by reference costs from a non-patient-specific source [12]. Gross costing is faster and cheaper but may lead to low accuracy because of the relatively large measurement units. Micro-costing is more reliable but may be expensive and not always practical [11].

Non-breast cancer controls were included in some studies. The costs among patients often incorporate some costs incurred jointly with other diseases or interventions, leading to the overestimation of the disease-specific costs. By comparing costs of breast cancer cases to control groups without breast cancer, breast cancer-attributable treatment costs can be estimated.

Description of mean costs by stage was reported in all studies. Some presented only point estimates, while others also reported the uncertainty of mean values, such as standard errors and confidence intervals.

Different regression models have been developed for cost modelling to approach the issues of cost data, such as the skewness, zero-values, and censoring [13]. In general, in cases of no censoring and no zero-costs, the log-gamma generalised linear model (GLM) is favoured, which deals with non-normality and avoids back-transformation issues [14]. Regarding the zero-cost issues, the two-part mixed model is the most informative by showing the possibility of any expenditure first. For the censoring issues, a regression model can be used which is weighted by the probability of not being censored. There is no unique model that can deal with all the problems, and the final choice depends on the type and design of the study.

Missing data could reduce the representativeness of samples and therefore distort inferences about the population. So we summarised the methods of dealing with missing data in the reviewed studies. Also, we assessed whether cost calculations were adjusted for inflation or any other changes.

## Results

### Search results

**Fig 1:**
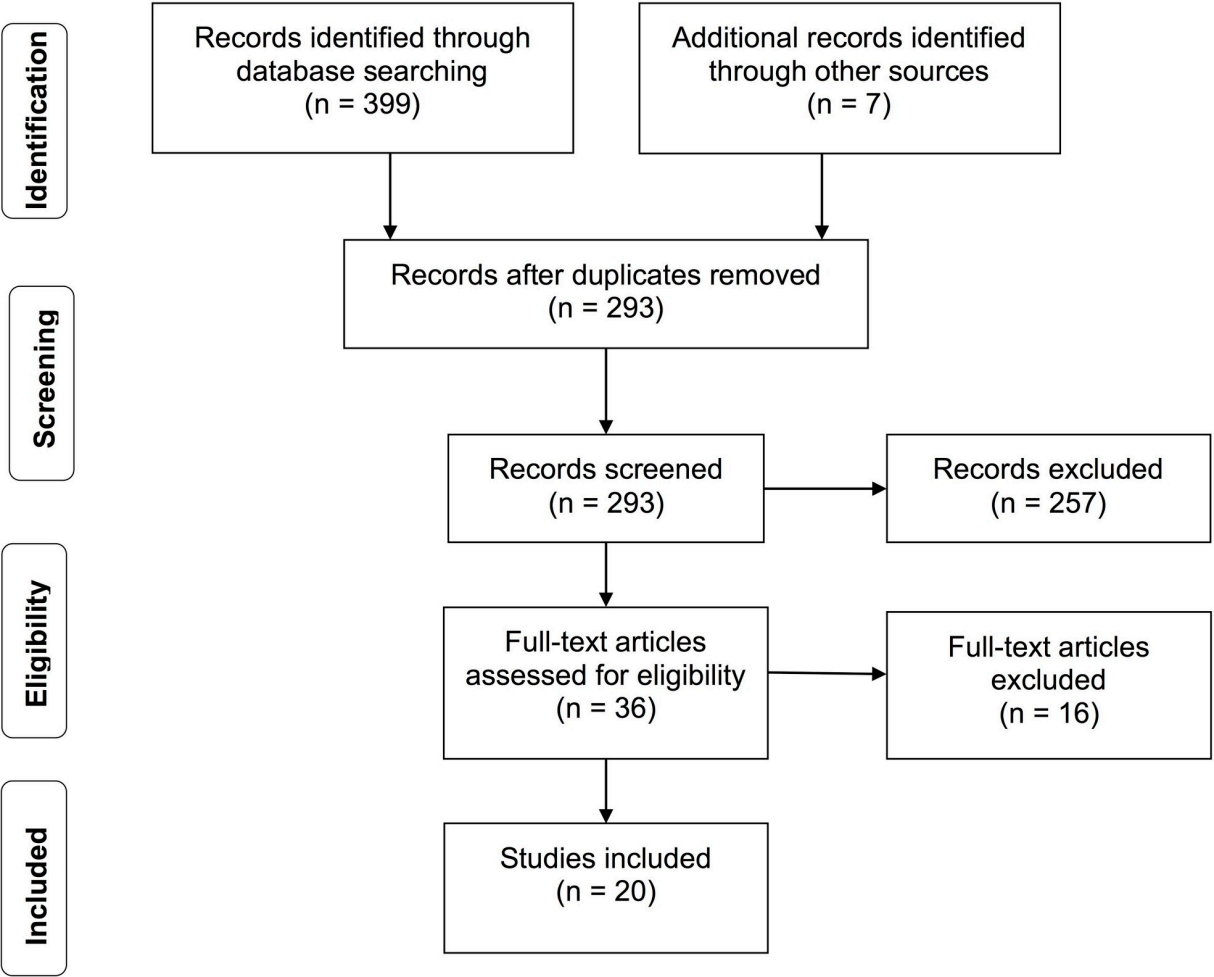
Study flow diagram.

The search took place in April 2018. MEDLINE search yielded 99 possible studies, EMBASE yielded 268, NHS EED yielded 32, and hand-searches produced seven from reference lists. The collective searches yielded 293 unique studies after removing duplicates. Based on the eligibility criteria, we excluded 273 studies and included 20 studies in this review (Fig 1). The two reviewers were in complete agreement for study eligibility. The identified studies were from ten countries: the US (n = 9), Canada (n = 2), China (n = 2), Italy (n = 1), Portugal (n = 1), the United Kingdom (UK) (n = 1), Vietnam (n = 1), France (n = 1), Iran (n = 1) and Mexico (n = 1).

### Treatment costs of breast cancer by stage

**Fig 2:**
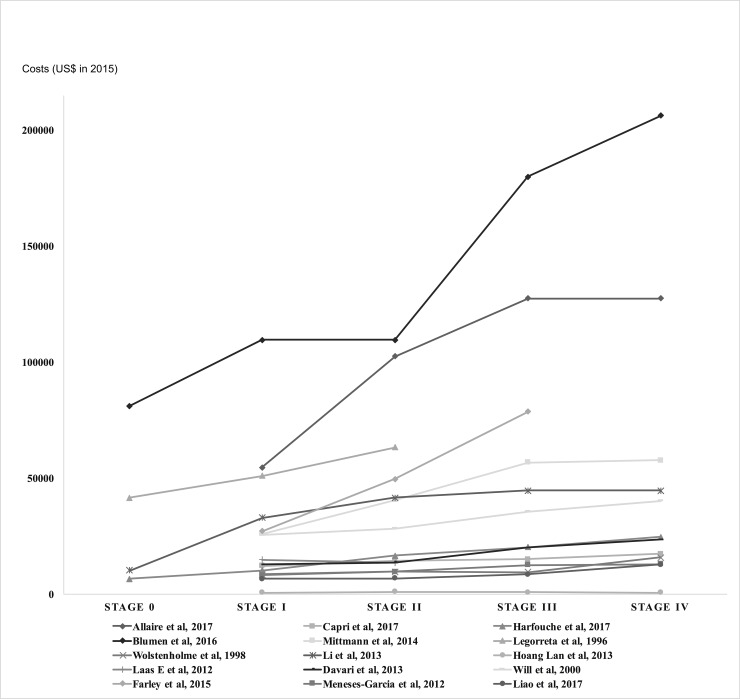
Cumulative breast cancer treatment costs by FIGO stages.

**Fig 3:**
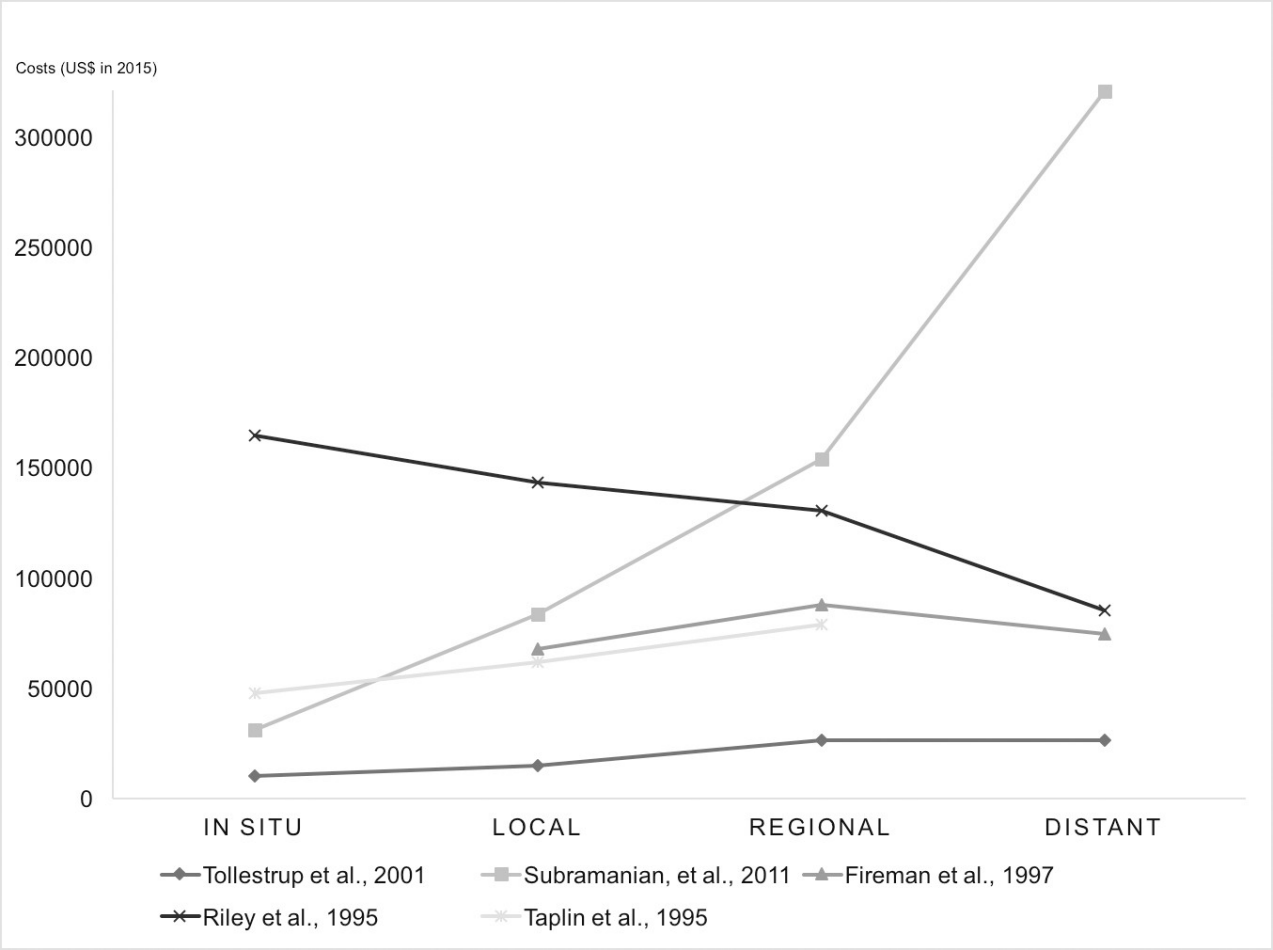
Cumulative breast cancer treatment costs by stages of in situ, local, regional and distant.

Table 1 summarises basic characteristics and cumulative treatment cost estimates by stage reported in the reviewed studies, with 85% agreement by two reviewers. Among the 15 studies using the FIGO staging system, the means of cumulative treatment costs weighted by sample sizes were $29,724 at stage I, $39,322 at stage II, $57,827 at stage III, and $62,108 at stage IV in 2015 US dollars. On average, costs at stage II, III and IV were 32%, 95%, and 109% higher than costs at stage I. In the other five studies where invasive breast cancer was categorised as local, regional and distant, the cost means weighted by sample sizes were $63,664, $89,898 and $168,906. Treatment costs of regional and distant breast cancer were 41% and 165% higher than local breast cancer on average. Figs 2 and 3 show that mean treatment costs generally increased with advanced stage at diagnosis.

**Table 1:** Basic characteristics and cumulative breast cancer treatment costs by stage (US dollars in 2015).

Study	Setting	Sample	Year	Time horizon	Costs by stage				
					0	I	II	III	IV
Allaire et al, 2017 [15]	US	4,082	2003–2010	1y ad^1^	-	54,664	102,528	127,444	
Capri et al, 2017 [16]	Italy	12,580	2007–2011	2y ad^1^	-	12,187	14,541	15,108	17,339
Harfouche et al, 2017 [17]	Portugal	807	2014	2y ad^1^	6,564	10,380	16,667	20,257	24,758
Blumen el al, 2016 [18]	US	8,360	2010	2y ad^1^	81,181	109,582		180,001	206,207
Mittmann et al, 2014 [19]	Canada	39,655	2005–2009	2y ad^1^	-	25,969	40,676	56,703	57,794
Wolstenholme el al, 1998 [20]	UK	137	1991	4y ad^1^	-	8,638	9,652	9,459	15,918
Legorreta el al, 1996 [21]	US	200	1989–1993	4y ad^1^	41,546	50,998	63,308	-	-
Li el al, 2013 [22]	China	316	2009–2010	5y ad^1^	10,296	32,884	41,632	44,595	44,766
Hoang Lan el al, 2013 [23]	Vietnam	160	2001–2006	5y ad^1^	-	654	1,038	939	694
Laas E et al, 2012 [24]	France	62	2010	Ac^2^	-	14,817	13,553	-	-
Will el al, 2000 [25]	Canada	17,700	1995	lifetime	-	25,755	28,392	35,628	40,212
Farley el al, 2015 [26]	US	274	2008–2010	Unk^3^	-	27,288	49,680	78,670	-
Davari el al, 2013 [27]	Iran	467	2005–2010	Unk^3^	-	12,838	13,734	20,035	23,643
Meneses-Garcia el al, 2012 [28]	Mexico	633	2004	Unk^3^	-	8,146	9,819	12,586	12,988
Liao et al, 2017 [29]	China	2,746	2012–2014	Unk^3^	-	6,706	6,794	8,556	12,840
					**In situ**	**Local**	**Regional**	**Distant**	
Tollestrup el al, 2001 [30]	US	317	1990–1994	1y ad^1^	10,219	14,824	26,502		
Subramanian el al, 2011 [31]	US	848	2002–2004	2y ad^1^	31,033	83,455	154,145	320,655	
Fireman el al, 1997 [32]	US	886	1987–1991	15y ad^1^	-	67,778	87,921	74,616	
Riley el al, 1995 [33]	US	Unk^3^	1973–1989	lifetime	164,727	143,367	130,472	85,128	
Taplin el al, 1995 [34]	US	2,944	1990–1991	Unk^3^	47,783	61,985	78,814	-	

Ad^1^ indicates after diagnosis, ac^2^: after chemotherapy, unk^3^: unknown.

The study by Riley et al. was not considered when we calculated the weighted mean values due to the unknown sample size [33]. This study reported that the lifetime payments between diagnosis and death were higher for patients diagnosed at an earlier stage, due to higher costs corresponded to longer survival time. However, they found that the annual average costs for patients diagnosed at earlier stages were substantially lower than annual costs at advanced stages. This supported the finding that earlier diagnosis lowers treatment costs.

We should be cautious when synthesising these treatment costs because the time horizons in the reviewed studies are different. Therefore, we also compared the cumulative treatment costs during the same time horizons. Four studies reported two-year cumulative treatment costs after diagnosis by FIGO stages among breast cancer patients. After conversion to 2015 US dollars, the costs estimated by Blumen et al. in the US [18] are much higher than the costs estimated in Italy [16], Portugal [18], and Canada [19]. The participants in Blumen’s study were commercially insured population, and they probably sought for more health services than populations with publically funded insurance.

Two other studies estimated five-year cumulative costs after diagnosis, with the study in China [22] reporting higher costs than the study in Vietnam [23]. The costs of breast cancer treatment in Vietnam were much lower than those reported in other countries, due to the limited use of new medications and advanced medical equipment during the study period [23]. The lack of affordable access to appropriate treatment of breast cancer also contributes to the low treatment costs. Some patients did not complete their treatment courses because they were not covered by insurance. In addition, the unit costs can be different across countries, such as the differences in remuneration of health staff and capital depreciation [23].

Two studies also reported the treatment costs at four years after diagnosis. The costs estimated by Legorreta et al. in the US [21] were higher than those estimated by Wolstenholme et al. in the UK [20]. However, both studies were conducted about thirty years ago and hence they were not very informative for the present comparison.

### Critical appraisal and methodological assessment

The quality of reviewed studies is presented in Table 2, as indicated by the percentage score ranging from 50% to 84%. Studies by Hoang Lan et al. [23] and Fireman et al. [32] had relatively high total scores among the reviewed papers. Studies scored relatively poorly on data collection items compared to other items. In addition, the choice of regression model was generally rarely justified. Table 3 summarises other aspects of the methodological assessment, with detailed study-specific results provided in S2 Table.

**Table 2:** Critical appraisal scores (percentages of maximum attainable scores).

Studies	Scored domains			Summary scores
	Study design	Data collection	Analysis and interpretation	
Allaire et al, 2017 [15]	6 (100%)	6 (38%)	10 (63%)	22 (58%)
Capri et al, 2017 [16]	6 (100%)	9 (56%)	8 (50%)	23 (61%)
Harfouche et al, 2017 [17]	6 (100%)	8 (50%)	8 (50%)	22 (58%)
Blumen, et al., 2016 [18]	5 (83%)	7 (44%)	8 (50%)	20 (53%)
Mittmann, et al., 2014 [19]	6 (100%)	6 (38%)	16 (100%)	28 (74%)
Wolstenholme et al., 1998 [20]	4 (67%)	8 (50%)	16 (100%)	28 (74%)
Legorreta et al., 1996 [21]	4 (67%)	9 (56%)	16 (100%)	29 (76%)
Li, et al., 2013 [22]	6 (100%)	12 (75%)	10 (63%)	28 (74%)
Hoang Lan et al., 2013 [23]	6 (100%)	10 (63%)	16 (100%)	32 (84%)
Laas E et al, 2012 [24]	6 (100%)	14 (88%)	9 (56%)	29 (76%)
Will, et al., 2000 [25]	4 (67%)	6 (38%)	14 (88%)	24 (63%)
Farley, et al., 2015 [26]	6 (100%)	7 (44%)	6 (38%)	19 (50%)
Davari et al., 2013 [27]	6 (100%)	6 (38%)	15 (94%)	27 (71%)
Meneses-Garcia el al, 2012 [28]	5 (83%)	6 (38%)	14 (88%)	25 (66%)
Liao et al, 2017 [29]	6 (100%)	8 (50%)	7 (44%)	21 (55%)
Tollestrup et al, 2001 [30]	5 (83%)	10 (63%)	14 (88%)	29 (76%)
Subramanian et al, 2011 [31]	5 (83%)	10 (63%)	8 (50%)	23 (61%)
Fireman et al, 1997[32]	5 (83%)	9 (56%)	16 (100%)	30 (79%)
Riley et al, 1995 [33]	5 (83%)	6 (38%)	12 (75%)	23 (61%)
Taplin et al, 1995 [34]	5 (83%)	9 (56%)	12 (75%)	26 (68%)
Average (Kappa = 0.69)	5.4 (89.2%)	8.3 (51.9%)	11.8 (73.4%)	25.4 (66.8%)

**Table 3:** Methodological assessment of the reviewed studies: frequency and percentage.

**Charges/claim**	Billed charges	Claim data	Unknown			
	9 (45%)	10 (50%)	1 (5%)			
**Cost collection**	Micro costing	Gross costing				
	15 (75%)	5 (25%)				
**Control groups**	Yes	No				
	6 (30%)	14 (70%)				
**Descriptive analysis**	Only mean	Mean and uncertainty				
	6 (30%)	14 (70%)				
**Regression models**	Parametric	Tobit	Two-part	GLM	Quantile	None
	6 (30%)	1 (5%)	1 (5%)	1 (5%)	1 (5%)	10 (50%)
**Censored data**	Described	Described				
	1 (5%)	19 (95%)				
**Missing data**	Imputation	CCA^1^	Assumption		Not mentioned	
	1 (5%)	9 (45%)	1 (5%)		9 (45%)	
**Timing issues**	Yes	No				
	16 (80%)	4 (20%)				

CCA^1^ indicates complete case analysis.

#### Charges/claims

Among the identified studies, nine studies used billed charges to measure costing data [20, 22, 23, 27–30, 32, 34], ten used claim datasets[15–19, 21, 24, 26, 31, 33], and one study did not provide information about this [25].

#### Cost collection and control groups

Fifteen studies used the micro-costing approach to measure and value cost [15–20, 23–25, 27, 28, 30–33]. However, they did not report the quantities of resource use separately from the unit costs. Five studies used the gross-costing approach to collect data [21, 22, 26, 29, 34]. Six studies included control groups to estimate the breast cancer-attributable treatment costs [15, 19, 30–32, 34].

#### Descriptive analysis

All of the reviewed studies estimated the means of breast cancer treatment costs in descriptive analyses. Fourteen studies among these also reported the uncertainty of estimated means [15, 19, 20, 22–24, 27–34], such as standard errors, 95% confidence intervals, or ranges between the minimum and maximum values.

#### Regression models

Ten studies used regression models to analyse the breast cancer treatment costs [16, 20–24, 30–32, 34]. Common parametric tests were conducted in six studies. Fireman et al. [32] used ordinary least squares (OLS) to analyse the relationship between patient characteristics and treatment costs. Three studies by Legorreta et al. [21], Wolstenholme et al. [20], and Li et al. [22] used analysis of variance (ANOVA) to examine differences in estimated costs across stages at diagnosis. Legorreta et al. [21] also used Chi-square test to evaluate the association between disease stage at diagnosis and other covariate variables. Taplin et al. [34] conducted multivariable regression for analysis, but the details of the models used were not explained.

Parametric approaches may sacrifice robustness when the assumptions of normality or homoscedasticity are violated. To deal with the large mass of observations with zero costs, Subramanian et al. [31] used the two-part model. In the first part, a logistic regression was conducted to predict the possibility of any expenditure. In the second part, the generalised linear model with a gamma distribution and a log link was used conditional on having positive expenditures. Tollestrup et al. [30] considered a Tobit regression model which allowed a point mass at zero but assumed an underlying normal distribution [35]. Also, Capri et al. [16] used a generalised linear model to identify predictors of log-transformed costs. In addition to the estimation of mean costs, Hoang Lan et al. [23] used the quantile regression aiming at estimating the conditional medians of costs.

#### Censored data

Meneses-Garcia el al. performed analysis of censored-data costs though no details were described [28]. In the other 19 studies, there was no mention to the approaches used to deal with censored observations.

#### Missing data

Only one study dealt with missing data by multiple imputation [20]. Nine studies conducted complete-case analysis [16, 17, 19, 23, 26–29, 34] and another made assumptions about the incomplete information [24]. In the remaining studies, there was no mention to the issue of missing data.

#### Timing issues

Sixteen studies considered timing issues such as using consumer price index (CPI) for inflation or discounting the cost values to reflect time preferences. In the other four studies [18, 22, 26, 28], timing issues were not described.

## Discussion

This study systematically reviewed published studies on breast cancer treatment costs by stage at diagnosis using patient level data from countries at different levels of socio-economic development. The review highlighted the fact that published data on this topic are rather limited and predominantly from high-income countries, and among the latter predominantly from the US. Of the 20 eligible studies identified, nine were from the US and only five from low- and middle-income countries. In addition, many of the studies were very dated. The paucity of the published evidence reflects in part the limited availability of staging information. The WHO International Classification of Diseases (ICD), which is the international standard used for reporting diseases and health conditions in routinely collected data, does not include codes for the stage at cancer diagnosis. Therefore, acquisition of stage information usually requires the collection of additional data from other (non-routine) sources or needs to be inferred from recommended stage-specific treatment protocols, neither of which is always feasible. It is worthwhile noting that the present review excluded studies that used a combination of clinical guidelines and unit costs, instead of patient level data, to estimate treatment costs as such cost estimates cannot reveal between-patient heterogeneity [36]. The review also excluded any data published in the grey literature by design, e.g. governmental reports, as its search was restricted to the scientific peer-reviewed literature. As studies with unfavourable results are less likely to be published, publication bias can be a potential concern for this review.

Nevertheless, the review’s findings are consistent with treatment costs increasing with the advancement of the disease stage at diagnosis. The mean treatment costs of stages II, III and IV breast cancer were 32%, 95%, and 109% higher than those of stage I disease, and the mean treatment costs of regional and distant breast cancer were 41% and 165% higher than those of local disease. It has been shown that patients with more advanced disease receive more treatments than early-stage patients, such as chemotherapy and targeted therapy [37]. Also, medication therapy is usually a costly part of treatment for patients at stages III and IV because of the prescription of more expensive drugs [27, 38, 39].

The review revealed between-country differences in treatment costs, with these likely to be partly due to the variation in treatment patterns. For example, the UK used human epidermal growth factor receptor 2 (HER2)-targeted medicine the least frequently among five European Union countries [40]. The US uses three times as many mammograms compared to other developed countries [41]. Also, the high administrative costs and drug costs in the US make the breast cancer treatment costs higher there than in many other high-income countries. Between-country differences in treatment costs might have also arisen because breast cancer survival rates vary widely across countries, overall ranging from 80% or over in North America to around 60% in middle-income countries and below 40% in low-income countries [42]. This reflects partly differences in stage at diagnosis as well as variations in the availability and access to appropriate treatments.

The review also revealed within-country variations in the treatment costs of breast cancer for the two countries with more than one study, i.e. the US, Canada, and China. Such differences may be partly due to differences in the years covered, i.e. the years of breast cancer diagnosis, as well as variations in time horizons. Advances in medicine have led to temporal changes in therapy strategies for breast cancer. Nowadays, breast conservation is the intended surgical standard approach for most women with early breast cancer [43]. Also, more systematic therapies have become available, such as endocrine therapy for hormone receptor positive breast cancer and target therapy for HER2-positive breast cancer [44].

The methodological assessment of the reviewed studies highlighted key methodological concerns. First, studies that used micro-costing approaches to collect cost data did not report the quantities of resources separately from the unit costs. Second, regression frameworks varied across studies. Some used common parametric tests such as ANOVA and OLS regression for cost estimations; however, these tests could be inappropriate due to the violation of their underlying assumptions. Two-part model and Tobit regression were conducted in some studies to deal with the impact of zero values, generalised linear model was applied to handle skewness, and quantile regression was used to estimate the median of costs. But the choice of these regression models was not fully justified. Third, only one study considered censored data though no details were described [28]. Censored data can be caused by death, loss to follow up, and administrative censoring [13]. If censoring is not accounted, the assessment of the importance of the disease severity on the cost of treatment may be biased [45]. Finally, the large majority of studies did not include a control group. Failure to do so might have resulted in an overestimation of cost values attributable to breast cancer treatment as some of the included costs might have been incurred by treatment of other co-existing diseases. All these methodological issues should be better dealt with in future costing studies.

## Conclusions

This systematic review highlighted the paucity of published studies on breast cancer treatment costs by stage, based on patient-level data, from both high-income and low/middle-income countries. Nevertheless, the limited available data are consistent with earlier detection of breast cancer being associated with lower treatment costs. More up-to-date studies on treatment costs of breast cancer by stage are required from beyond the US including other developed and developing regions. Further costing studies should properly address and clearly describe key methodological issues (e.g. skewness, zero values, censored data, missing data).

## Supporting information

S1 Checklist PRISMA Checklist. (DOC)

S1 Table Search strategy. (DOCX)

S2 Table Detailed methodological assessment of the reviewed studies. (DOCX)
