# Supplementary material for: Global treatment costs of breast cancer by stage: A systematic review
Source: PLoS One. 2018 Nov 26;13(11):e0207993. doi: 10.1371/journal.pone.0207993 (PMC6258130; doi:10.1371/journal.pone.0207993)
Supplement: S1 Table — (DOCX) [file pone.0207993.s002.docx]

**S1 Table**

|  | Searches | MEDLINE | EMBASE | NHS EED |
| --- | --- | --- | --- | --- |
| 1 | Breast cancer or breast tumor or breast tumour or breast neoplasm or mammon cancer or mammo tumor or mammo tumour or mammo neoplasm | 208573 | 485451 | 2043 |
| 2 | exp Breast Neoplasms/ | 262672 | 482860 | 1798 |
| 3 | 1 or 2 | 298094 | 529150 | 2339 |
| 4 | Cost or treatment cost or health service cost or drug cost or surgery cost or mastectomy cost or breast-conserving cost or chemotherapy cost or radiotherapy cost or endocrine cost or targeted therapy cost | 378394 | 762229 | 22534 |
| 5 | exp Health Care Costs/ | 57935 | 263288 | 4990 |
| 6 | exp Health Expenditures/ | 19239 | 263288 | 213 |
| 7 | 4 or 5 or 6 | 409040 | 776053 | 23053 |
| 8 | 3 and 7 | 6009 | 15490 | 793 |
| 9 | Disease stage or cancer stage or by stage or stage-specific | 23486 | 39578 | 318 |
| 10 | Local and regional and remote | 3781 | 6807 | 4 |
| 11 | I and II and III and IV | 63321 | 104261 | 255 |
| 12 | 9 or 10 or 11 | 89546 | 148561 | 549 |
| 13 | 8 and 12 | 99 | 268 | 36 |
