# Supplementary material for: Global treatment costs of breast cancer by stage: A systematic review
Source: PLoS One. 2018 Nov 26;13(11):e0207993. doi: 10.1371/journal.pone.0207993 (PMC6258130; doi:10.1371/journal.pone.0207993)
Supplement: S2 Table — (DOCX) [file pone.0207993.s003.docx]

**S2 Table**

| Study | Type | Collection | Match | Description | Regression | Censoring | Missing data | Timing |
| --- | --- | --- | --- | --- | --- | --- | --- | --- |
| Allaire et al, 2017 [15] | Claim | Micro | Yes | Mean, UNC^2^ | -- | No | NM | Yes |
| Capri et al, 2017 [16] | Claim | Mirco | No | Mean | GLM | No | CCA^3^ | Yes |
| Harfouche et al, 2017 [17] | Claim | Mirco | No | Mean | -- | No | CCA^3^ | Yes |
| Blumen et al, 2016 [18] | Claim | Micro | No | Mean | -- | No | NM | No |
| Mittmann, et al, 2014 [19] | Claim | Micro | Yes | Mean, UNC^2^ | -- | No | CCA^3^ | Yes |
| Wolstenholme et al, 1998 [20] | Charge | Micro | No | Mean, UNC^2^ | ANOVA | No | CCA^3^, impute | Yes |
| Legorreta et al, 1996 [21] | Claim | Gross | No | Mean | χ2, ANOVA | No | NM^4^ | Yes |
| Li et al, 2013 [22] | Charge | Gross | No | Mean, UNC^2^ | ANOVA | No | NM^4^ | No |
| Hoang Lan et al, 2013 [23] | Charge | Micro | No | Mean, UNC^2^ | Quantile | No | CCA^3^ | Yes |
| Laas E et al, 2012 [24] | Claim | Micro | No | Mean, UNC^2^ | χ2, Fisher | No | Assumption | Yes |
| Will et al, 2000 [25] | UNK^1^ | Micro | No | Mean | -- | No | NM^4^ | Yes |
| Farley et al, 2015 [26] | Claim | Gross | No | Mean | -- | No | CCA^3^ | No |
| Davari et al, 2013 [27] | Charge | Micro | No | Mean, UNC^2^ | -- | No | CCA^3^ | Yes |
| Meneses-Garcia el al, 2012 [28] | Charge | Micro | No | Mean, UNC^2^ | -- | Yes | CCA^3^ | Yes |
| Liao et al, 2017 [29] | Charge | Gross | No | Mean, UNC^2^ | -- | No | CCA^3^ | Yes |
| Tollestrup et al, 2001 [30] | Charge | Micro | Yes | Mean, UNC^2^ | Tobit | No | NM^4^ | No |
| Subramanian et al, 2011 [31] | Claim | Micro | Yes | Mean, UNC^2^ | Two-part | No | NM^4^ | Yes |
| Fireman et al, 1997 [32] | Charge | Micro | Yes | Mean, UNC^2^ | OLS | No | NM^4^ | Yes |
| Riley et al, 1995 [33] | Claim | Micro | No | Mean, UNC^2^ | -- | No | NM^4^ | Yes |
| Taplin et al, 1995 [34] | Charge | Gross | Yes | Mean, UNC^2^ | Multivariate | No | CCA^3^ | Yes |

UNK^1^ indicates unknown, UNC^2^: uncertainty, CCA^3^ indicates complete case analysis, NM^4^: not mentioned.
